# Exosome microsphere/nano silver loaded injectable antibacterial hydrogel augments anti-infection and healing for scald wound

**DOI:** 10.3389/fmicb.2025.1550276

**Published:** 2025-06-25

**Authors:** Rongkai Li, Qinbing Qi, Xiaojing Jiang, Zhongfei Gao, Xianrui Xie, Yuqing Zhao, Xiaochen Cheng, Chunhua Wang, Guige Hou, Chengbo Li

**Affiliations:** ^1^School of Pharmacy, Key Laboratory of Medical Antibacterial Materials of Shandong Province, Binzhou Medical University, Yantai, China; ^2^Biomedical Laboratory, School of Medicine, Liaocheng University, Liaocheng, China

**Keywords:** exosomes, microsphere, nano sliver, antibacterial hydrogel, scald treatment

## Abstract

Infectious scald is recognized as a life-threatening concern in clinical due to easy infection and hard recovery. Exosomes possess cell migration, proliferation and angiogenesis under the containing rich growths factors, which may be a possibility for the treatment of scald chronic wounds. However, the instability and release issues of exosomes limits the storage and therapeutic efficacy. Herein, in this study, exosomes were successfully encapsulated calcium alginate microspheres, effectively avoiding the leakage and inactivation of exosomes. Meanwhile, composite hydrogel (HS-QAF-AgNPs) was fabricated with and quaternized carboxymethyl chitosan (Q-CMC), aldehyde group-modified alginate (ASA) with assisted crosslinking of ferric ion and dopped nano silver (AgNPs). The incorporation of exo-CAMs into the hydrogel protects the exosomes from harsh environmental conditions and extends their diffusion time, thereby guaranteeing a better therapeutic effect. Q-CMC and AgNPs altogether endow the hydrogel system with an inherent antibacterial effect against pathogenic microorganisms. *In vitro* and *in vivo* investigations demonstrated that the HS-QAF-AgNPs hydrogel was cytocompatibility, possessed antibacterial and hemostatic properties, promoted cell migration and cell proliferation, stimulated angiogenesis, regulated inflammatory response, delayed apoptosis, enhanced collagen deposition and accelerated wound closure. Thus, the multifunctional HS-QAF-AgNPs hydrogel dressing is expected to be a promising candidate for the treatment of severe scalds. There is an urgent need to develop multifunctional wound dressings that can coordinate cell migration, proliferation, apoptosis, collagen formation and remodeling, alongside inflammation and angiogenesis to promote skin wound closure.

## 1 Introduction

Skin is one of body’s primary multifunctional tissues, which serves as a protective barrier against external mechanical aggressions, invading pathogens, and sensing external stimuli (de Jong et al., 2010). Scald wounds, resulting from exposure to hot liquids or steam, have become a common cause of skin trauma (Fu et al., 2024). Inadequately care can lead to infections, which could finally cause organ failure in multiple systems, disability, and in the most severe cases, death. Currently, wound dressings containing antibiotics are primarily used to treat infected wounds, however, this approach often contributes to the development of drug-resistant bacterial strains (Zhou et al., 2018; Sun et al., 2024). Additionally, the effectiveness of frequently prescribed antibiotics is significantly reduced due to the emergence of drug-resistant bacteria (Klein et al., 2019). Herein, exploring novel therapeutic strategies aimed at enhancing scald infected wound healing and tissue regeneration is an extremely urgent requirement.

Recently, innovative wound dressings such as electrospun scaffolds, sponges and hydrogels have been developed and fabricated to enhance wound closure and promote healing (Sathiyaseelan et al., 2017; Gao et al., 2021; Ding et al., 2024; Koohzad and Asoodeh, 2024). Among the various biomaterials, hydrogel dressings, particularly injectable self-healing hydrogel dressings, are considered to be promising candidates for the treatment of both acute and chronic wounds. Given their unique structures, a wide range of natural polysaccharides, such as starch, alginate, chitosan, cellulose, and hyaluronic acid, have been extensively utilized in the fabrication of injectable self-healing hydrogel dressings (Eskandarinia et al., 2019; Zhang and Zhao, 2020; Cao et al., 2022; Long et al., 2022; Yuan et al., 2023).

Over past few decades, regenerative medicine has emerged as a promising strategy for tissue repair and regeneration (Rani Raju et al., 2022). Human umbilical cord-derived mesenchymal stem cells (hucMSCs) possess advantageous biological properties that are highly beneficial for skin regeneration and wound healing (Shaikh et al., 2022). Exosomes derived from hucMSCs (hucMSCs-exos) have been widely used in the treatment of chronic wounds (Teng et al., 2022). Recently, exosome-based hydrogels for the wound healing has been raised (Shiekh et al., 2020; Bakadia et al., 2023). Nonetheless, despite their positive therapeutic efficacy, exosomes still face challenges, such as instability and deficiency of antimicrobial properties, which hinder clinical applications. Another significant challenge lies in ensuring the control release of exosomes over an extended period of time following their passive encapsulation into hydrogels (Shi et al., 2017; Li et al., 2020). How to better apply exosomes in combination with multifunctional hydrogels has been urgent issues to be develop functional dressings for treating infectious scald wounds. Recent advancements in hydrogel-based exosome delivery systems have primarily focused on optimizing sustained release mechanisms for regenerative and therapeutic applications. Engineered hydrogels, such as alginate and hyaluronic acid, encapsulate exosomes through physical, covalent, or affinity-based interactions, thereby enabling controlled release over periods ranging from days to weeks. These systems enhance tissue repair (like cardiac and cartilage regeneration), promote wound healing, and improve cancer therapy by preserving the bioactive cargo of exosomes (like miRNAs and proteins) while minimizing their rapid clearance from the body. Innovations in this field include the development of stimuli-responsive hydrogels that provide spatiotemporal precision and synergistic therapeutic effects, such as enhanced angiogenesis in diabetic ulcers. Despite promising results in preclinical studies, challenges remain regarding scalability, standardization of exosome production, precise control of hydrogel degradation, and the establishment of clinical-grade manufacturing processes. Key applications of these systems encompass tissue engineering, chronic disease management, and immunomodulatory therapies (Zhou et al., 2022; Han et al., 2024; Liu et al., 2024).

**SCHEME 1:**
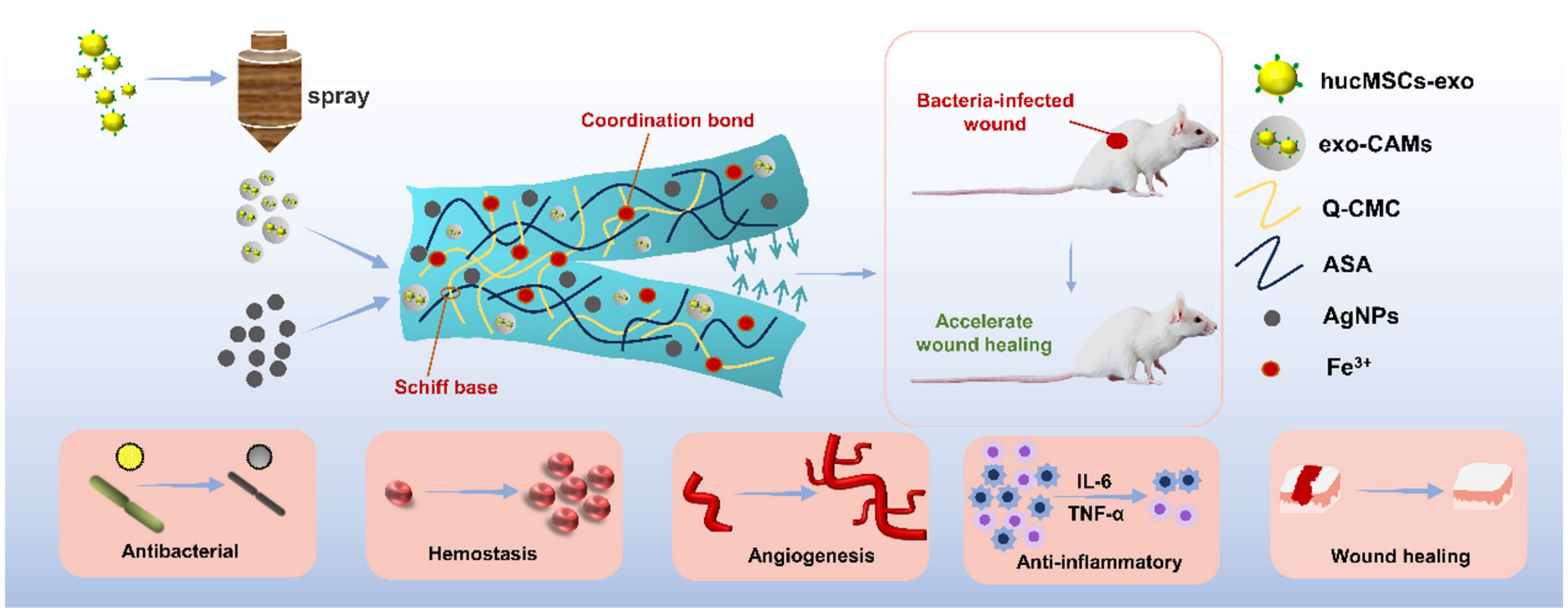
Schematic diagram of process of HS-QAF-AgNPs composite hydrogel and applications in infected scald wound healing.

Based on our previous work (Li et al., 2023), we designed an injectable and self-healing HS-QAF-AgNPs hydrogel aimed at accelerating the healing of infectious scald wounds (Scheme 1). Initially, the hucMSCs-exos were embedded into calcium alginate microspheres to maintain their biological activity. We then fabricated a new class of multifunctional hydrogel based on the resultant (exo-CAMs), dodecyl quaternary ammonium salt-modified carboxymethyl chitosan (Q-CMC), aldehyde group-modified sodium alginate (ASA), Fe^3+^ and silver nanoparticles (AgNPs) via Schiff bases and coordination bonds. The calcium alginate microspheres protect hucMSCs-exos against the hostile environment. The incorporation of exo-CAMs into hydrogels prolongs the exosome diffusion time and enhances therapeutic effect. The addition of Ag^+^ and grafting of positive charge are profoundly effective approaches to synergize antimicrobials. The structure of HS-QAF-AgNPs hydrogel was characterized and its rheological behavior was further assessed. Additionally, the hemostatic properties, antibacterial effects and cytotoxicity were estimated by relative tests. Moreover, the healing rate, collagen deposition, angiogenesis, and anti-inflammatory effects of HS-QAF-AgNPs hydrogel were evaluated by infected scald wound model. The proposed HS-QAF-AgNPs hydrogel dressing is expected to be a promising candidate for the treatment of infected skin wounds.

## 2 Materials and methods

### 2.1 Materials

Carboxymethyl chitosan (Mn = 150–800 kDa) was purchased by Yuanye Bio-Technology Co., Ltd. (Shanghai, China) Yeast powder, trypsin and Sabouraud agar medium were obtained from OXOID (British). LPS and Dio Kit were provided by Sigma-Aldrich (USA). Nano Silver Solution (1,000 ppm, 2 nm) were provided by Aladdin Reagent Co., Ltd. (Shanghai, China). Sodium alginate [SA, Pharmaceutical Grade, Mw = 20–40 kDa, viscosity = 200 ± 20 mPa·s (1% aqueous solution at 20–25°C)] and huMSCs-exos were purchased from Shanghai Macklin Biochemical Technology Co., Ltd. (Shanghai, China). *Staphylococcus aureus* (ATCC6538), *Escherichia coli* (ATCC25922) and *Pseudomonas aeruginosa* (ATCC9027) were purchased from American Type Culture Collection. All mice were obtained from Shandong Pengyue Experimental Animal Technology Co., Ltd. Meanwhile, the Institutional Animal Ethics Committee of Binzhou Medical University, Yantai (protocol No. 2022–049) approved all the animal experiments.

### 2.2 Synthesis and characterizations of the exo-CAMs and HS-QAF-AgNPs hydrogel

The spray method was used to prepare exo-CAMs. Firstly, 1.5 wt% CaCl_2_ solution containing 50 μg/ml of hucMSCs-exos was sprayed evenly into 6% CaCl_2_ solution. The exo-CAMs were centrifuged and cryopreserved. Based on previous studies (Li et al., 2023), the QAF gel was employed as a carrier hydrogel for exo-CAMs and AgNPs. QAF was synthesized by crosslinking aldehyde-modified sodium alginate (ASA), quaternized carboxymethyl chitosan (Q12-CMC), and trivalent iron ions (FeCl_3_). PBS (0.1 M, pH 7.4) was used as the solvent. To prepare solution A, exo-CAMs (100 mg/ml) were dispersed in 5 ml of 5% ASA solution. For solution B, AgNPs (10 mg) were dispersed in 5 ml of 5% Q12-CMC solution. Solutions A and B were then mixed thoroughly by vertexing and homogenization. Subsequently, 1 ml of 1% FeCl_3_ solution was added to the mixture, followed by vertexing and incubation at 24°C for 10 min to form the composite gel HS-QAF-AgNPs.

Particle size distribution of the exo-CAMs was tested with a laser particle sizer (Zetasizer nanoZS, Malvern, England). The exo-CAMs were stained with Dio fluorescent dye, and the distribution and state of hucMSCs-exos in the microspheres were observed by laser confocal microscope (LSM880, Zeiss, Oberkohen, Batenwerburg, Germany). The morphology of exo-CAMs and HS-QAF-AgNPs hydrogels were evaluated by SEM microscope (EVO LS1503040702, Zeiss, Oberkohen, Batenwerburg, Germany). Morphological analysis was performed using a EVO LS15 of ZEISS at an accelerating voltage of 10 kV, working distance of 8 mm, and magnification of 50×. The human umbilical cord mesenchymal stem cell-derived exosomes were stained with DIO to label their membranes, followed by observation under a confocal microscope. Imaging was performed using a 488 nm laser wavelength to excite green fluorescence, with parameters set at 1024 × 1,024 pixels resolution and 30 × magnification. Samples were sputter-coated to ensure conductivity. EDX system (Inca X-Max20, Oxford, England) was used for elemental analysis.

### 2.3 Antibacterial activity

The antibacterial effectiveness of QAF and HS-QAF-AgNPs hydrogels was evaluated using the plate count method with *E. coli*, *S. aureus* and *P. aeruginosa*. Freeze-dried hydrogel samples (diameter 1 cm, thickness 5 mm) were placed in 24-well plate with sterile PBS to achieve adequate swelling and then exposed to a bacterial suspension of 10^7^ CFU/ml for 24 h. Subsequently, 100 μL of co-cultured bacterial solution was plated on LB agar and incubated at 37°C for 24 h. The shaking bed was used to simulate the wound condition, so that the bacteria and hydrogel were fully and evenly contacted to avoid local concentration deviation.

Bacterial suspensions were serially diluted (10-fold in PBS) and plated onto Mueller-Hinton agar. Plates were incubated aerobically at 37°C for 24 h. Colonies were counted manually using a colony counter (SCAN 300, Interscience, France). Untreated bacterial suspensions and sterile agar served as positive and negative controls, respectively. Each experiment was repeated three times. The reduction rates of bacteria were calculated by Equation 1.

(1) $$\text{Redution rate of bacteria}\;(\%)=\frac{q_{\text{control}}-q_{\text{sample}}}{q_{\text{sample}}}\times 100\%$$

Where the variables *q_control_* and *q_sample_* represent the colony counts for the blank control group and the experimental groups, respectively.

### 2.4 *In vivo* wound healing capacity

Forty-eight healthy 8-week-old male Balb/c mice (weighing 20–22 g) were randomly assigned to four groups, with four predetermined sampling time points. Consequently, three mice were allocated to each group at each time point. After transdermal preparation, II–IIIa-degree burn wounds were created on shaved back skin of anesthetized mice by exposing it to a round plastic tube filled with boiling water for 30 s. The dead skins (0.8 cm in diameter) were then removed and infected with *S. aureus* suspension for 18 h. The animals were treated with gauze (Control), Tegaderm (a commercial breathable film from 3 M company), QAF, and HS-QAF-AgNPs, respectively. Photos of the treated wound were collected with a digital camera on postoperative days 3, 7, 10, 14 and 21. The wound healing rate was calculated as Equation 2.

(2) $$\text{Wound healing rate}\;(\%)=\frac{S_{0}-S_{A}}{S_{0}}\times 100\%$$

Where *S_0_* is the original wound area on day 0, and *S_A_* is the wound area on day A.

Histological analysis was performed with paraffin sections using HE staining, Masson’s trichrome staining, immunofluorescence (IHC; CD31, α-SMA, Caspase3, CD86 and CD206) and immunohistochemistry (TNF-α and IL-10) to further verify the pathological changes and expression of related factors.

### 2.5 Data analysis

Statistical analysis was conducted using one-way ANOVA (α = 0.05) followed by Tukey’s *post hoc* test, which was selected to compare means across three or more independent groups. Data are expressed as mean value ± SEM (*p < 0.05, **p < 0.01, ***p < 0.001, ****p < 0.0001). All data statistics and analysis are done through GraphPad Prism v8.03 (GraphPad Software Inc., San Diego, CA, USA) and Image J (NIH, Bethesda, MD, USA).

## 3 Results and discussion

### 3.1 Characterizations of the exo-CAMs and HS-QAF-AgNPs hydrogel

The network of HS-QAF-AgNPs hydrogel was crosslinked with Schiff base between Q-CMC and ASA, and coordination bonds from Fe^3+^, which contained the encapsulated exo-CAMs and AgNPs. Based on a spray technique, hucMSCs-exos were first encapsulated in calcium alginate microspheres with sustainable bioactivity. The exo-CAMs, Q-CMC, ASA, Fe^3+^ and AgNPs were mixed, and HS-QAF-AgNPs hydrogels were finally prepared. Such a design strategy aimed to achieve following main advantages. Firstly, the antibacterial property was enhanced by Q-CMC and AgNPs. Secondly, the coordination bonds between carboxymethyl in Q-CMC and Fe^3+^ and the dynamic Schiff base contribute to the excellent self-healing property of the hydrogel. Thirdly, long effective duration was given by sustained exosomes release. The calcium alginate microspheres are physically embedded within the polymer matrix rather than chemically participating in the primary crosslinking network (Schiff base/Fe^3+^/AgNPs). Their incorporation may influence the gelation kinetics and mechanical properties of the resulting material. The presence of microspheres could potentially delay the crosslinking process by restricting the molecular mobility of polymer chains, while residual Ca^2+^ ions may weakly coordinate with alginate fragments within the matrix.

**Figure 1:**
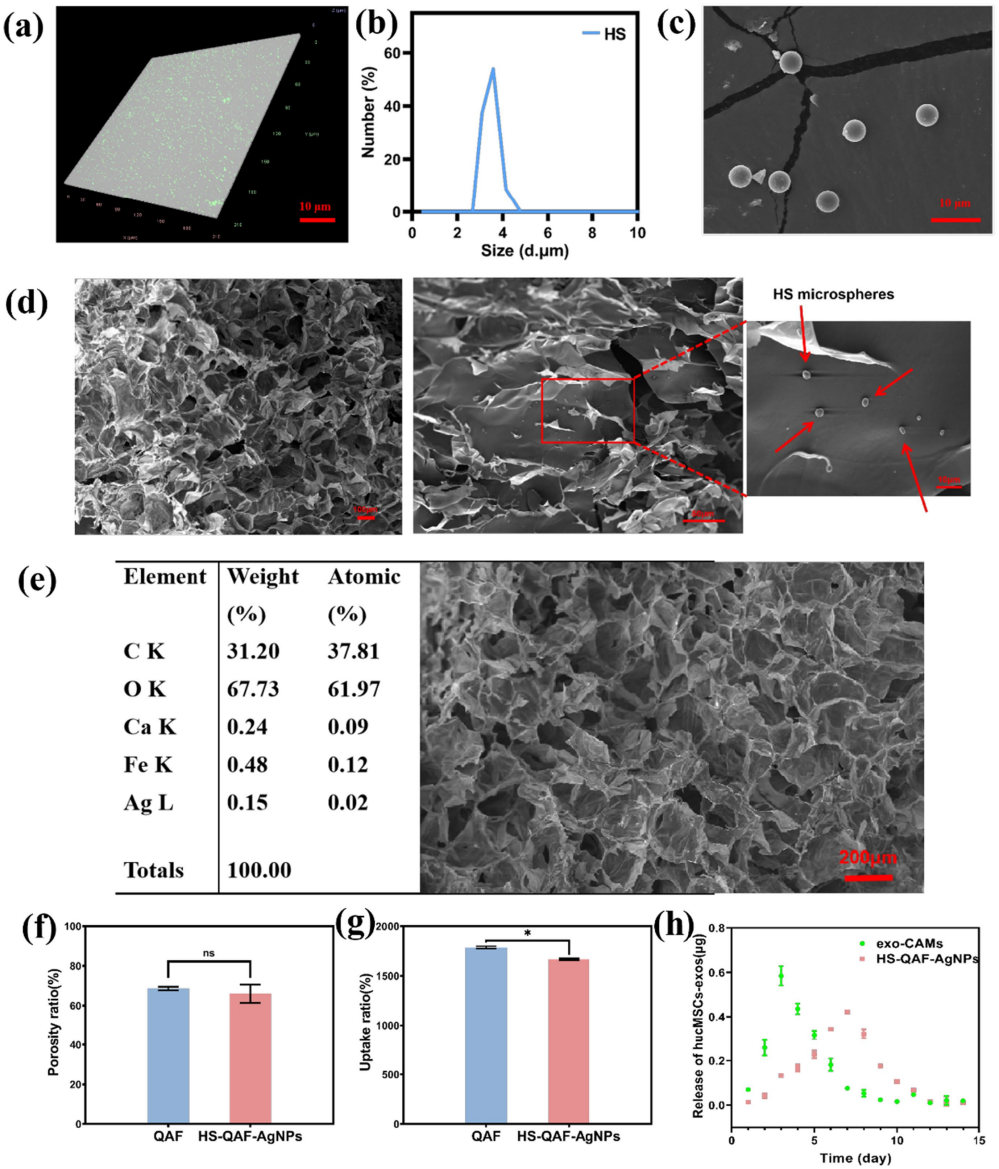
**(a)** LSCM images of exo-CAMs; **(b)** Particle size distribution of exo-CAMs; **(c)** Fluorescent images of exo-CAMs with Dio-labeled hucMSCs-exos; **(d)** SEM images of HS-QAF-AgNPs hydrogel and the red arrows show exo-CAMs distributed evenly in the hydrogel; **(e)** EDX analysis of HS-QAF-AgNPs hydrogel indicating the presence of Ca^2+^ and Ag; **(f)** The porosity of HS-QAF-AgNPs hydrogel and QAF hydrogel (*n* = 3; ns, not significant); **(g)** The water uptake properties of hydrogel (*n* = 3, *p < 0.05); **(h)** Releasing curve of hucMSCs-exos.

The morphology of exo-CAMs was observed using SEM. It was determined that spray method produced uniform microspheres with a diameter range from 3 to 4 μm (Figures 1a,b). Exosomes embedded in microspheres were labeled by Dio, a lipophilic dye for membrane labeling. Confocal fluorescence microscopy analysis demonstrated that the Dio-labeled hucMSCs-exos (green dots) successfully anchored within the calcium alginate microspheres (Figure 1c), indicating the suitability of microspheres as carriers for hucMSCs-exos. The hydrogel possessed a honeycomb structure with large pores, and the pore size distribution of the hydrogel was determined by SEM images, which ranged from 80 to 120 μm. Exo-CAMs were homogeneously distributed within the HS-QAF-AgNPs hydrogel (Figure 1d; Supplementary Figure S1). The EDX analysis of the HS-QAF-AgNPs hydrogels showed the distribution of calcium and silver, which proved that AgNPs and calcium alginate microspheres were successfully encapsulated in HS-QAF-AgNPs hydrogel (Figure 1e).

A significant challenge in the management of chronic wounds lies in maintaining an optimal balance of moisture during healing process (Aramwit et al., 2010; Xiao et al., 2021). A moist wound environment is crucial for promoting anti-inflammatory and angiogenesis, ultimately exerting beneficial effects in wound healing disorders. Hence, excellent wound dressings should have porosity to retain a humid environment and improve tissue regeneration. The optimal wound dressing should possess a high porosity ranging from 60 to 90% in order to facilitate cell infiltration and proliferation (Yin et al., 2020). The porosity of HS-QAF-AgNPs hydrogel was 65.97 ± 4.57% (Figure 1f), endowing hydrogels with rapid water uptake rate and 15 times dead-weight of uptake capacity (Figure 1g). The porosity of HS-QAF-AgNPs hydrogel was slightly lower than that of QAF hydrogel, attributed to the incorporation of exo-CAMs and AgNPs, resulting in a denser inner structure.

The release of hucMSCs-exos from exo-CAMs and HS-QAF-AgNPs hydrogel was detected (Figure 1h). The release kinetics of hucMSCs-exos in exo-CAMs indicated that hucMSCs-exos exhibited a sustained release for 7 days with a burst release at 3rd day. The burst release of hucMSCs-exos could be attributed to the degradation of exo-CAMs (Bao et al., 2017; Jing et al., 2021). The hucMSCs-exos released from HS-QAF-AgNPs hydrogel showed an initial burst release within the first 7 days, followed by a gradual release over the next 5 days. The double-layer embedding of exo-CAMs and HS-QAF-AgNPs hydrogel showed sustained release of hucMSCs-exos for 12 days. These findings indicated that the HS-QAF-AgNPs hydrogel had the potential to serve as an effective delivery system for exosomes release in order to enhance wound healing.

### 3.2 Injectable, self-healing property of the HS-QAF-AgNPs hydrogel

**Figure 2:**
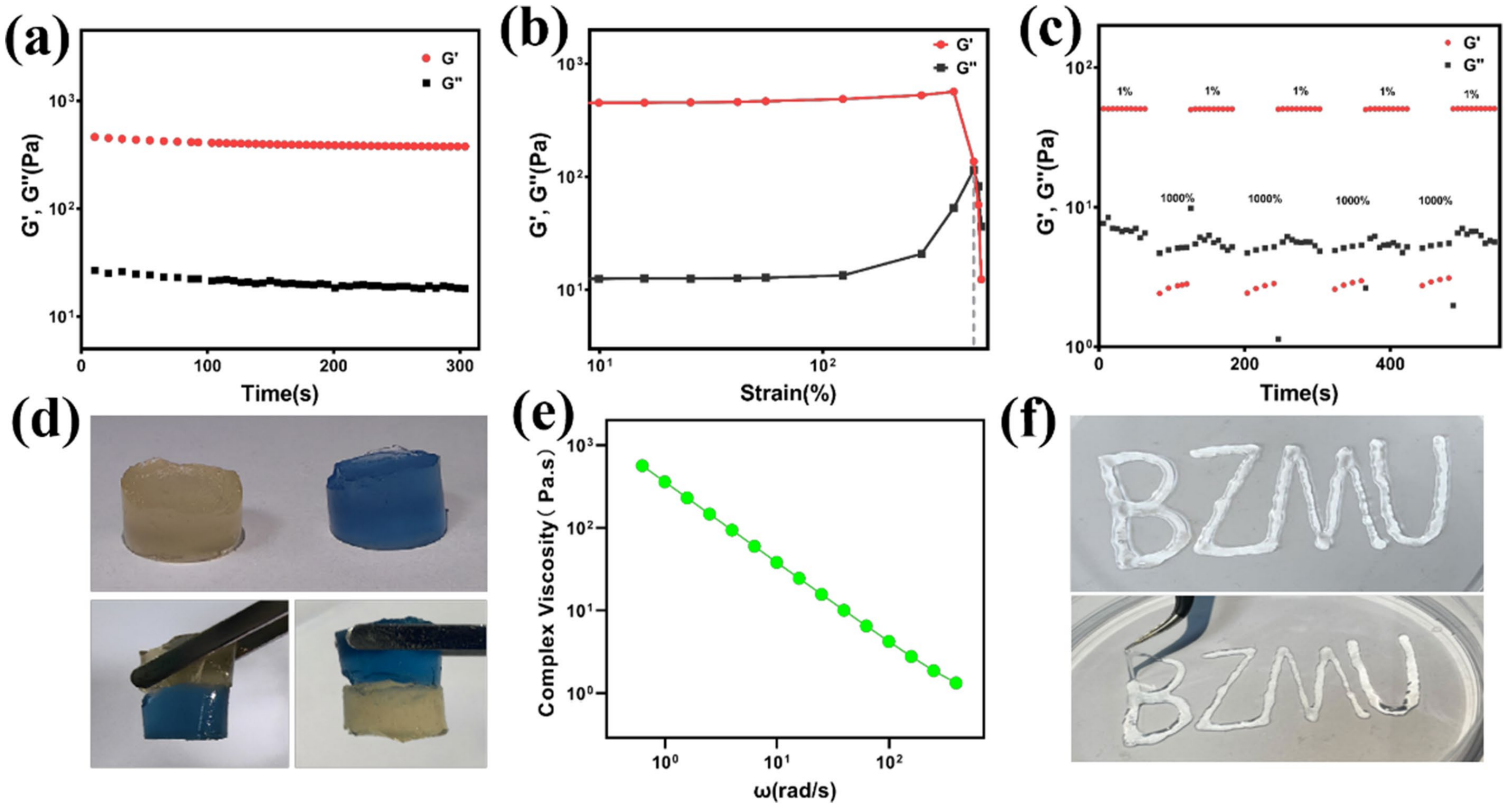
**(a)** Strain amplitude sweep test of HS-QAF-AgNPs hydrogel; **(b)** Continuous step strain test of HS-QAF-AgNPs hydrogel; **(c)** Rheological properties of the hydrogel when alternate step strain was switched from 1 to 1,000%; **(d)** Photographs of HS-QAF-AgNPs hydrogel macroscopic self-healing performance; **(e)** The viscosity of HS-QAF-AgNPs hydrogel; **(f)** Injectable property of HS-QAF-AgNPs hydrogel.

Hydrogel dressings is susceptible to damage from external forces, but self-healing hydrogels have the ability to repair themselves, thereby prolonging the lifespan of the dressing (Yang et al., 2022). The rheological properties of HS-QAF-AgNPs hydrogel, including storage modulus (G′) and loss modulus (G″) were investigated. The results showed that G′ was bigger than G″ for HS-QAF-AgNPs hydrogel, indicating their gel nature at 25°C (Figure 2a; Ke et al., 2020). The amplitude sweeping test curve for HS-QAF-AgNPs hydrogel in Figure 2b showed that HS-QAF-AgNPs hydrogel exhibited a broad linear viscoelastic region and a great anti-shear ability. To assess the self-healing properties, a continuous step strain experiment was carried out, and results were depicted in Figure 2c. The hydrogel demonstrated exceptional self-healing capability, as evidenced by both rheological recovery and macroscopic healing assessments. Upon exposure to increasing strain (1 to 1,000%), the storage modulus (G′) significantly decreased below the loss modulus (G″), indicating network disruption caused by the rupture of reversible crosslinks. Nevertheless, upon reverting to 1% strain, G′ promptly recovered to its original value, thereby confirming the dynamic and adaptive nature of the crosslinked network. Macroscopically, when the hydrogel was severed into two distinct pieces, the fractured surfaces spontaneously re-fused within 2 h without external intervention (Figure 2d). The healed hydrogel regained sufficient mechanical integrity to withstand manipulation with tweezers. This self-healing behavior was ascribed to the presence of dynamic reversible interactions, including Schiff base linkages and metal-coordination bonds (Zhao et al., 2018; Yu et al., 2023), which facilitated bond reformation following rupture. Shear thinning experiment indicated disruption in its crosslinked structure and resulting in injectable behavior (Figure 2e). As indicated in Figure 2f, HS-QAF-AgNPs hydrogel could easily be injected through needles, resulting in thin filaments. These data proved that the hydrogels had excellent injectability and self-healing capabilities.

### 3.3 Evaluation of antibacterial properties

**Figure 3:**
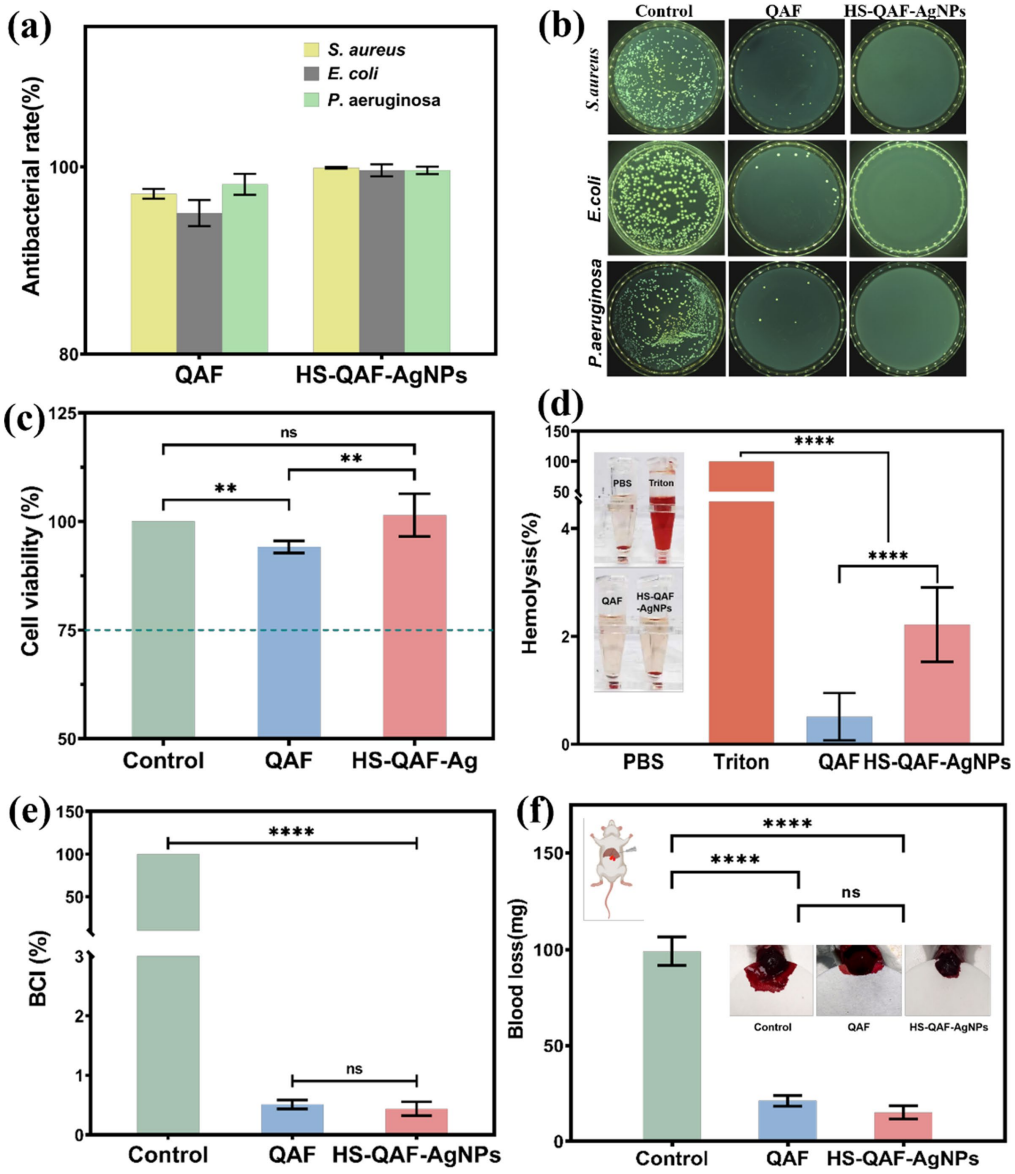
Antibacterial properties of hydrogels. **(a)** The counter board pictures of *S. aureus*, *E. coli* and *P. aeruginosa* growth on QAF and HS-QAF-AgNPs hydrogels; **(b)** Antibacterial rates of QAF and HS-QAF-AgNPs hydrogels, *n* = 3; **(c)** Viability of L929 cells incubated with the hydrogel extracts by CCK-8 assay, *n* = 3; **(d)** Statistical hemolysis ratio of Triton, PBS, QAF hydrogel and HS-QAF-AgNPs hydrogel, *n* = 3 (Insert: Hemolytic photographs); **(e)** The BCI values of whole blood clotting evaluation, *n* = 3; **(f)** Statistics of blood loss without treatment or with hydrogel treatments, *n* = 3 (Insert: Schematic diagram of the liver incision model) *p < 0.05, **p < 0.01, **p < 0.001, ****p < 0.0001.

Bacterial contamination can lead to serious consequences, such as poor skin regeneration, chronic wound, septicemia and even death (Jiang et al., 2023; Qi et al., 2023). A suitable antibacterial wound dressing is supposed to isolate the external bacterial and effectively eliminate excessive bacterial infection at wound site. The antibacterial activity of HS-QAF-AgNPs hydrogel was studied to fight againist *S. aureus*, *E. coli*, and *P. aeruginosa*. Compared to the blank group (PBS), the bactericidal efficiency of HS-QAF-AgNPs hydrogel towards *S. aureus*, *E. coli*, and *P. aeruginosa* is basically >99% (Figure 3a). The above results were consistent with results in Figure 3b. For the QAF hydrogel, Fe (III) and quaternary ammonium group jointly exert antibacterial activity by disrupting the cell membrane via electrostatic interactions with the bacterial surface. In this study, the addition of AgNPs exhibited higher antibacterial performances with inhibition rate of more than 99% than QAF. Thus, HS-QAF-AgNPs hydrogel showed good inherent antibacterial activity, because doped nano-silver can induce redox reactions within bacterial structures, thereby causing multi-faceted bacterial inactivation (Cheng et al., 2013; Gollapudi et al., 2024). The HS-QAF-AgNPs hydrogel exhibited superior bactericidal ability compared to the QAF hydrogel, suggesting that the quaternary ammonium salt groups and silver nanoparticles exerted a synergistic enhancement on the antibacterial effect.

### 3.4 Hydrogel cytotoxicity and hemocompatibility measurement

Incorporating sterilizing ingredients into wound dressing can effectively prevent wound infection, while these may cause cytotoxicity and diminish the therapeutic efficacy. The potential toxicity of the hydrogel was assessed by exposing L929 fibroblast cells to the leachate of HS-QAF-AgNPs. Further, CCK-8 assay was used to exam cell proliferation and the results were shown in the Figure 3c. Cell morphology and growth density of HS-QAF-AgNPs hydrogels were significantly greater than those of QAF groups, indicating the introduction of hucMSCs-exos was in favor of promoting cell adhesion and proliferation (Xiong et al., 2022).

Hemocompatibility test was performed to inspect the biosafety. The macroscopical color of centrifugally obtained supernatants and there was no significant hemolysis in HS-QAF-AgNPs hydrogel group. The hemolysis rate of HS-QAF-AgNPs hydrogel was approximately four times higher than that of QAF hydrogel (Figure 3d). The application of silver nanoparticles is limited due to their dose-dependent hemolytic effects. However, the hemolysis rate of HS-QAF-AgNPs hydrogel was 2.22 ± 0.69%. Moreover, the gradual release of minimal amounts of AgNPs throughout the self-healing hydrogel can effectively reduce potential cell toxicity of Ag. As shown in Figure 3e, the hemostatic performance of QAF and HS-QAF-AgNPs hydrogel were assessed through *in vitro* coagulation test. Hydrogels exhibited significantly reduced BCI values compared to the control group. Compared to exist reports on AgNPs loaded hydrogel (Makvandi et al., 2019; Huang et al., 2022b), exosome microspheres can potentially enhance biocompatibility by delivering multiple growth factors and promoting the synthesis of extracellular matrix, thereby improving tissue regeneration and integration. For example, exosomes may enable more precise antibacterial distribution and enhance cell adhesion via bio-membrane fusion. Moreover, the phospholipid bilayer structure of exosomes can minimize non-specific interactions with red blood cells. In summary, this study demonstrates that the synergistic design of exosomes and nano-silver not only maintains high antibacterial efficiency but also exhibits superior biocompatibility compared to nano-silver gels alone.

Additionally, HS-QAF-AgNPs hydrogel demonstrated a lower BCI value than QAF hydrogel, possibly attributed to the presence of silver inducing denaturation of anticoagulant proteins and impacting the intrinsic pathway of blood coagulation by shortening clotting time (Madhumathi et al., 2010). We further evaluated the hemostatic ability of HS-QAF-AgNPs hydrogel in mouse liver incision bleeding models (Figure 3f). The model data indicated that both hydrogel treatments demonstrated a clear hemostatic effect. Mice in the control group (without treatment) experienced approximately 98.97 ± 7.16 mg of blood loss in the liver incision, while those treated with QAF and HS-QAF-AgNPs hydrogel showed reduced blood loss to 21.13 ± 2.74 and 15.09 ± 3.44 mg, respectively.

### 3.5 *In vitro* cell migration and tube formation

**Figure 4:**
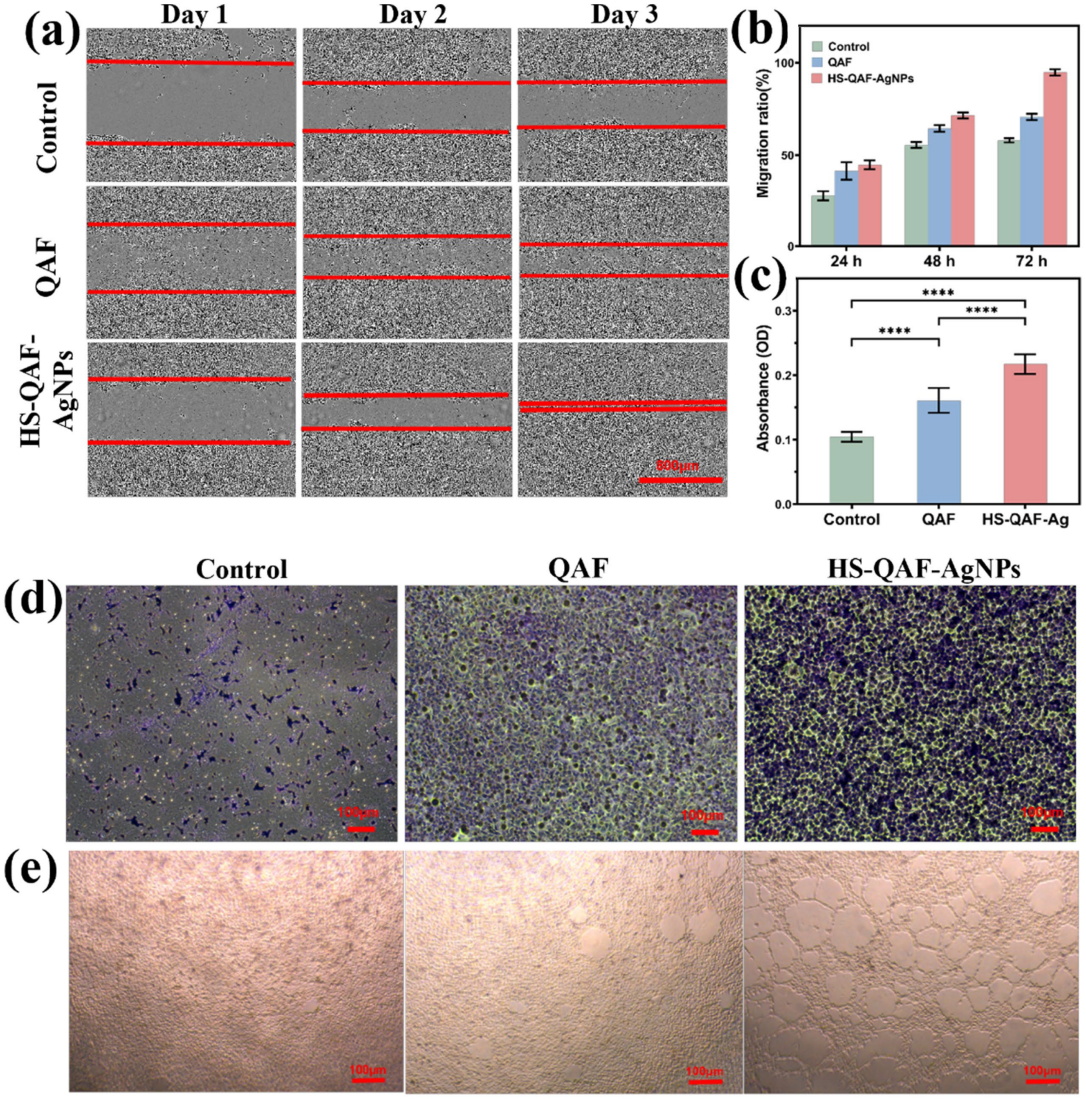
**(a)** Cell migration assay after cultured for 1, 2, and 3 days. **(b)** Quantitative analysis of cell migration after 1, 2, and 3 days (*n* = 3). Quantitative analysis **(c)** and representative images **(d)** of the relative migrated percentage of Hacats cells from the transwell assay (*n* = 3, ****p < 0.0001). **(e)** The tube formation assay after incubation with HS-QAF-AgNPs hydrogel.

HS-QAF-AgNPs hydrogel is expected to offer excellent biological properties that facilitate cellular migration, proliferation, and tissue regeneration. HaCat was utilized for cell scratch assays and Transwell assays. After cultured for 2 to 3 days, migration ratios of the HS-QAF-AgNPs group were approximately 71.44 ± 1.70% and 94.75 ± 1.70%, respectively. Cell scratch assays showed that the HS-QAF-AgNPs group significantly enhanced scratch closure compared to the QAF group and control group (Figures 4a,b). Transwell assays results demonstrated that the proposed hydrogels significantly enhanced cell migration. Compared to the untreated controls, migrated HaCat cells were increased by 2.1-fold in HS-QAF-AgNPs hydrogel groups (Figures 4c,d).

The stimulation and acceleration of the angiogenic activity is a major requirement for enhancing the infectious scald wound healing (Han et al., 2022). Substantial evidence have shown that hucMSCs-exos play an important role in angiogenesis by mediating cell-to-cell communication (Sahoo et al., 2011; Bian et al., 2014). The proangiogenic effect of HS-QAF-AgNPs hydrogel was evaluated HUVECs cells’ angiogenesis. As shown in Figure 4e, HS-QAF-AgNPs hydrogel treatment markedly enhanced the tubule formation capacity. With the introduction of exo-CAMs and AgNPs, the tube number and branch length increased significantly compared to the QAF hydrogel (Supplementary Figure S2). Exosome-derived vascular endothelial growth factor (VEGF) modulates endothelial cell function via paracrine signaling mechanisms. Upon fusion of VEGF with the exosomal membrane, it is specifically targeted and delivered to the surface of endothelial cells, thereby inducing cyclin expression, which in turn promotes endothelial cell proliferation and migration (Cui et al., 2020). Furthermore, the synergistic interaction between VEGF and miRNAs enhances functional outcomes by upregulating the expression of tight junction proteins, ultimately facilitating angiogenesis and tube formation (Chen et al., 2019). Additionally, recent studies show that silver nanoparticles (AgNPs) enhance angiogenesis and reduce oxidative stress through multiple mechanisms: Ag^+^ released from AgNPs upregulates VEGF and HIF-1α, activating the PI3K/Akt/mTOR pathway to promote endothelial cell migration and tubulogenesis (Wu et al., 2020). Additionally, their surface plasmon resonance boosts superoxide dismutase (SOD) activity, lowering malondialdehyde (MDA) levels by 40% via ROS scavenging (Zhao et al., 2023).

### 3.6 *In vitro* anti-inflammation activity of hydrogel

**Figure 5:**
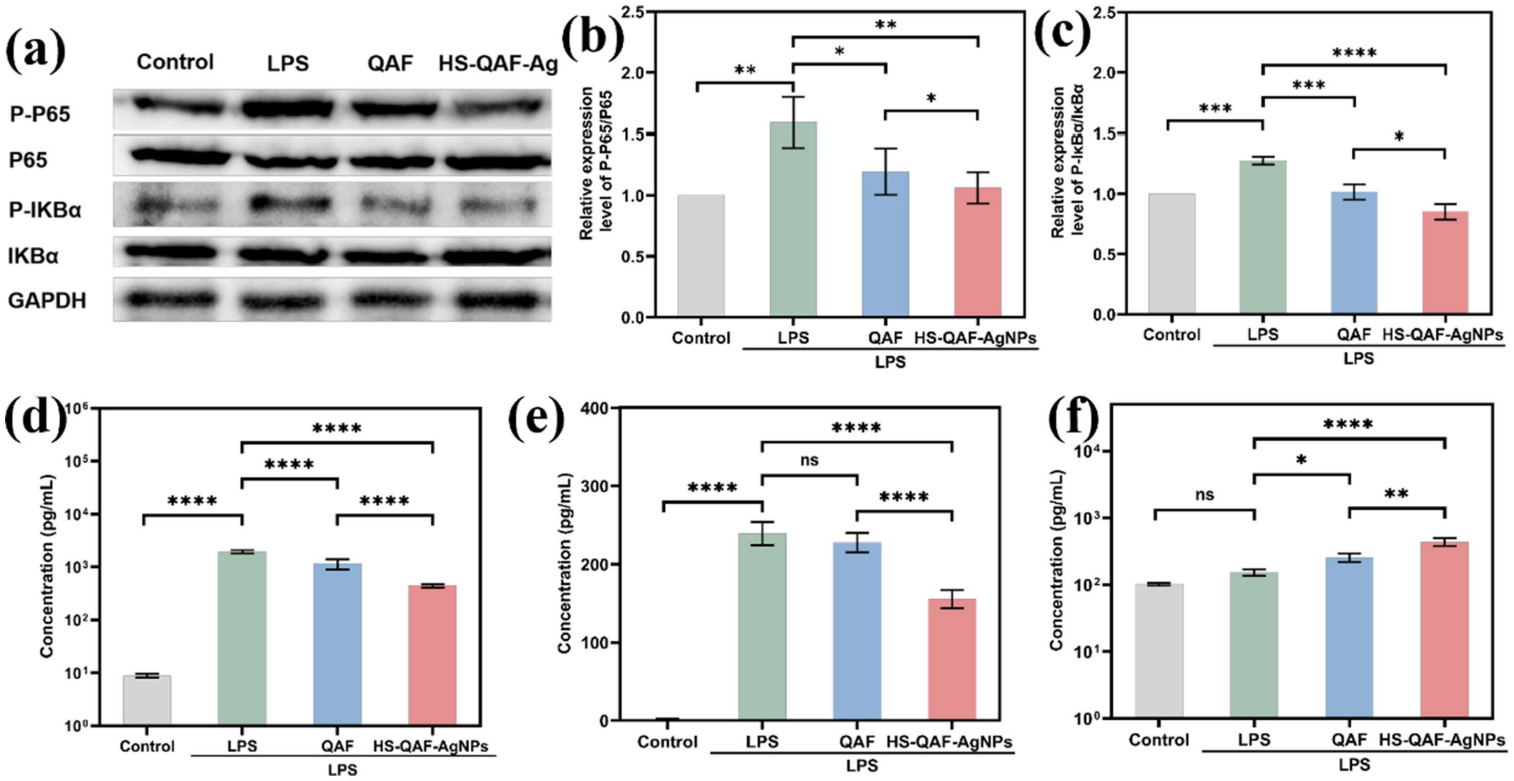
**(a)** Western Blot analysis of the P-P65, P65, P-IκB-α, IκB-α protein expression in each group; **(b)** Quantitative analysis of the P-P65, P65 expression in each group (*n* = 3); **(c)** Quantitative analysis of P-IκB-α, IκB-αexpression in each group (*n* = 3); **(d)** IL-6, **(e)** TNF-α and **(f)** IL-10 concentrations of RAW264.7 cells were treated with different hydrogel (*n* = 3; *p < 0.05, **p < 0.01, ***p < 0.001, ****p < 0.0001).

Chronic wound healing is a highly intricate biological process and excessive inflammatory infiltration can interfere with normal healing process (Huang et al., 2022a). Therefore, reducing excessive inflammation plays a crucial role in restoring the condition of the original skin. RAW264.7 cells were cultured on various hydrogels for 3 to 7 days following treatment with LPS to evaluate the anti-inflammatory efficacy. Phosphorylation status of NF-κB, p65 and IκB-α were quantified using western blotting. The results in Figures 5a–c revealed that the HS-QAF-AgNPs hydrogel downregulated LPS-induced expression of p-NF-κB, p65 and p-IκB-α, suggesting that the HS-QAF-AgNPs hydrogel might regulate inflammatory response by inhibiting the activation of NF-κB pathway. NF-κB pathways are involved in the regulation of proinflammatory cytokines, such as IL-6, IL-10 and TNF-α (Li and Verma, 2002; Liang et al., 2004). The expression levels of the pro-inflammatory cytokine (IL-6 and TNF-α) and anti-inflammatory cytokine (IL-10) were quantified using Elisa kit. As shown in Figures 5d–f, the concentration of IL-6 and TNF-α significantly increased after LPS treatment, with the IL-10 concentration significantly reduced compared with the control group. Furthermore, the concentration of the pro-inflammatory cytokines IL-6 and TNF-α were decreased and the concentration of the anti-inflammatory cytokines IL-10 increased following treatment with HS-QAF-AgNPs hydrogel. Suppression of the NF-κB pathway was consistent with the reduced levels of IL-6 and TNF-α and the increased level of IL-10 following treatment with HS-QAF-AgNPs hydrogel. These results suggested that the fabricated hydrogel suppressed pro-inflammatory cytokine expression and induced anti-inflammatory cytokine expression to regulate the inflammatory response in infectious scald wound.

### 3.7 *In vivo* wound healing

**Figure 6:**
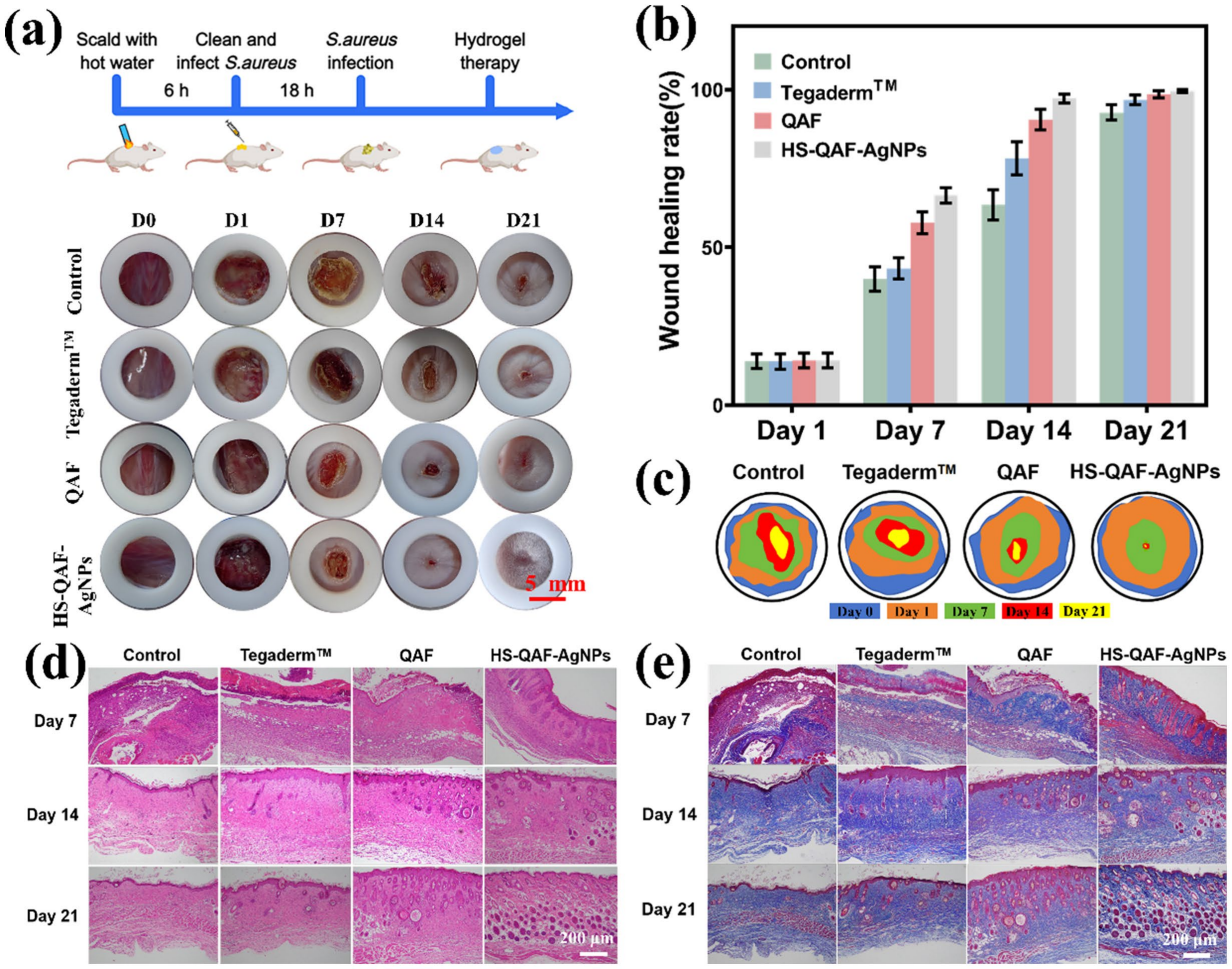
**(a)** Photographs of wound healing process at different time points. Wound healing rate **(b)** and schematic diagram of wound contour trend **(c)** after treated with different dressings (*n* = 3). **(d)** H&E staining of the wounds treated with different dressings. **(e)** Masson staining micrographs of the wounds treated by different dressings.

The healing-promoting abilities of HS-QAF-AgNPs hydrogels were evaluated in a mouse full-thickness defect infection model. Figure 6a showed the effects of HS-QAF-AgNPs, QAF, gauze and HS-QAF-AgNPs on days 7, 14 and 21. On day 7, all the wounds had regional tissue shrinkage with the different dressing treatment. The wounds treated with HS-QAF-AgNPs hydrogel showed obvious contraction. After 14 days post-treatment, the wound healing rate of the HS-QAF-AgNPs hydrogel group was significantly faster than that of other groups, because the hydrogel not only effectively killed bacteria, but also released exosomes at wound site to accelerate wound healing. After 21 days, the wounds in each group were basically healed, and the hydrogel-treated group were completely closed. As Figures 6b,c presented, the wound closure rate of Tegaderm™ film, QAF hydrogel and HS-QAF-AgNPs hydrogel treatments groups were 96.74 ± 1.47%, 98.50 ± 1.14% and 99.50 ± 0.43%. The repair effect of HS-QAF-AgNPs hydrogel group was the best, indicating that HS-QAF-AgNPs hydrogel was more conducive to infected wound repair after the addition of AgNPs and hucMSCs-exos. Loading nano silver enhances the antibacterial properties of the hydrogels, resulting in the effective bacteria-killing and prevents wound infection (Shanmugapriya et al., 2020). In addition, exosomes could regulate all phases of skin wound healing (Prasai et al., 2022). Overall, these results *in vivo* experiments showed that the HS-QAF-AgNPs hydrogels exhibited promising prospects for wound healing.

The treated tissues were stained by hematoxylin–eosin staining (H&E) to evaluate wound healing after days 7, 14, and 21. As shown in Figure 6d, the inflammatory reaction in the HS-QAF-AgNPs hydrogel group was slighter than in other groups on day 7, confirming that AgNPs-loaded hydrogels can effectively reduce wound inflammatory response. The HS-QAF-AgNPs hydrogel-treated group had lower inflammatory cell density, more hair follicles and displayed better vascular reconstruction on day 21. Masson’s trichrome staining was performed on the wound tissues of each group at various time points to evaluate collagen formation and remodeling at the wound site (Figure 6e). The collagen content of the HS-QAF-AgNPs hydrogel group was higher than control group, the Tegaderm™ group and the QAF hydrogel group. On day 21, the collagen fibers in HS-QAF-AgNPs hydrogel group were arranged in a tight and neatly manner, with highest collagen density, and the morphology of the fibers was closer to normal skin. Therefore, HS-QAF-AgNPs hydrogel could effectively promote collagen deposition.

NF-κB is a critical nuclear transcription factor that regulates physiological processes such as cellular growth and inflammatory responses. The HS-QAF-AgNPs hydrogel suppresses the NF-κB signaling pathway by releasing silver nanoparticles (AgNPs) and human umbilical cord mesenchymal stem cell-derived exosomes (hUC-MSC-exosomes), which target both the IκBα protein and the p65 subunit. The IκBα protein, a key signaling transducer, primarily inhibits NF-κB pathway activation by sequestering NF-κB complexes in the cytoplasm. AgNPs reduce oxidative stress by scavenging reactive oxygen species (ROS), thereby inhibiting the activation of the IκB kinase (IKK) complex and blocking IκBα phosphorylation. Concurrently, exosomal miRNAs exert targeted modulation to suppress p65 phosphorylation. Consequently, treatment with HS-QAF-AgNPs significantly reduces IκBα phosphorylation levels and decreases phosphorylated p65 protein, resulting in a synergistic effect that alleviates inflammatory responses at wound sites and accelerates healing.

### 3.8 Immunofluorescence and Immunohistochemical analysis

**Figure 7:**
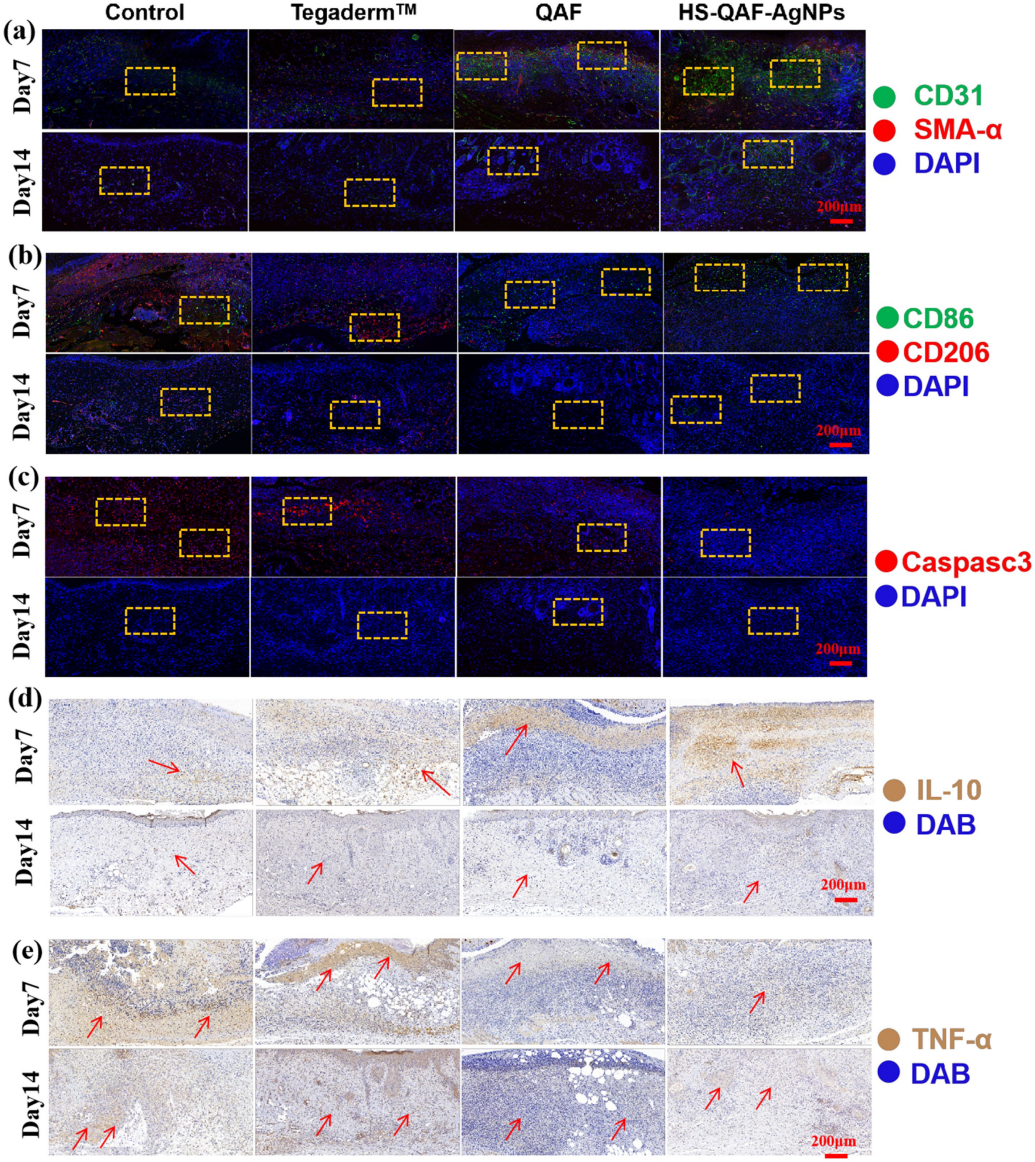
**(a)** CD31 and α-SMA, **(b)** CD86 and CD206, **(c)** Caspase-3 immunofluorescence on 7 and 14 days. **(d)** IL-10 and **(e)** TNF-α immunofluorescence staining of histological sections.

Neovascularization is a crucial process during wound healing. Insufficient angiogenesis may result in venous insufficiency, thereby impeding wound healing or leading to the formation of chronic wounds (Han et al., 2022). The expression of CD31 and α-SMA, was evaluated by IHC to assess angiogenesis content. According to Figure 7a, the HS-QAF-AgNPs hydrogel group exhibited significantly greater CD31 (green) and α-SMA (red) areas on days 7 and 14 compared to other groups, indicating a higher abundance of generated blood vessels.

To investigate the effect of HS-QAF-AgNPs hydrogel treatment on M1 and M2 macrophage development, wound samples were immunofluorescence staining with CD86 (a marker for M1 macrophages) and CD206 (a marker for M2 macrophages). As shown in Figure 7b, on the 7th day and 14th day, the HS-QAF-AgNPs hydrogel group showed the highest expression of CD86 and the lowest of CD 206 compared with other groups. Based on above results, HS-QAF-AgNPs hydrogel could avoid long-term inflammation dominated by M1 macrophages and promote M2 macrophage development during wound healing. Caspase-3 (red) were selected as indicators to evaluate the apoptosis of transplanted skin tissue. As shown in Figure 7c, it was evident that the HS-QAF-AgNPs hydrogel effectively suppressed cell apoptosis.

Immunofluorescence images were annotated highlight regions of interest. Contrast adjustments were applied uniformly across all images to ensure visibility of labeled structures without altering the original data.

Cytokines play a crucial role in regulating the initiation and communication of cellular process associated with tissue repair (Munir et al., 2019). We evaluated the expression of cytokines to explore the mechanism of HS-QAF-AgNPs dressing in promoting wound healing. In Figures 7d,e, the HS-QAF-AgNPs hydrogel group had the least amount of TNF-α and the largest amount of IL-10 compared with other groups, demonstrating that the HS-QAF-AgNPs hydrogel could effectively alleviate excessive inflammatory responses caused by infection. In conclusion, the HS-QAF-AgNPs hydrogel groups showed a decrease in the release of pro-inflammatory factors and an increase in the release of anti-inflammatory factors, which showed a more effective repair effect compared to the control group.

This study conducted preliminary investigations at the *in vitro* cellular level and on skin defects, and significant further development is required before clinical application can be realized. While some nano-silver-containing products have been commercialized, their use remains limited to local anti-infection applications due to relatively high toxicity. The toxicity of nano-silver primarily arises from the sustained release of silver ions, which may lead to accumulation *in vivo*. In this study, sodium alginate microspheres were employed as carriers for exosomes. On one hand, this approach mitigates issues of degradation and burst release associated with prolonged exosome exposure. On the other hand, exosomes enable precise delivery of nano-silver to infection sites via surface markers (like CD63), thereby reducing systemic toxicity risks. Nano-silver and exosomes were co-encapsulated into an antibacterial hydrogel to address most microbial infections and promote tissue repair, offering a potential strategy for sequential treatment of chronic infections in clinical practice. However, extensive mechanistic studies and technological advancements are necessary prior to clinical implementation. For example, physical and chemical interactions between exosomes and nano-silver (such as electrostatic adsorption or membrane fusion) might influence the release of exosomal contents or the antibacterial activity of nano-silver, necessitating further investigation into structural stability post-co-incubation. Additionally, the risk of *in vivo* accumulation resulting from long-term combined use of nano-silver and exosomes requires further evaluation. Furthermore, the hydrogel used in this study serves only as a prototype drug carrier and lacks microenvironment-responsive properties. Developing suitable dosage forms or intelligent responsive materials capable of achieving “on-demand release” at lesion sites instead of traditional “slow release” could potentially reduce the *in vivo* risks associated with nano-silver usage.

The nano-silver/exosome composite hydrogel facilitates wound healing through precise spatiotemporal regulation. During the inflammatory phase, nano-silver suppresses the NF-κB signaling pathway via the release of Ag^+^ ions, thereby reducing the expression levels of TNF-α and IL-6 and accelerating the resolution of inflammation (Chen et al., 2023). In the proliferative phase, exosomes deliver VEGF to enhance endothelial cell migration (Chen et al., 2023), while nano-silver catalyzes superoxide dismutase (SOD) activity to decrease reactive oxygen species (ROS) levels at the wound site (Lei et al., 2025), synergistically promoting angiogenesis. During the remodeling phase, active factors within exosomes regulate fibroblast behavior, inducing the formation and reorganization of collagen fibers. Simultaneously, nano-silver mitigates matrix degradation, contributing to the orderly alignment of collagen fibers. This dynamic spatiotemporal regulation not only shortens the inflammatory phase but also minimizes scar formation and accelerates the regeneration of functional tissue.

## 4 Conclusion

In this work, an exosome microsphere and nano silver-loaded composite hydrogel, HS-QAF-AgNPs was prepared by one-step mixing method to explore its therapeutic efficacy for infected scald wound. The HS-QAF-AgNPs hydrogel showed advantageous properties, including efficient self-healing capabilities, excellent injectability, high water absorption, good biocompatibility, and efficient hemostatic performance. Synergistic of quaternary ammonium salt group and AgNPs endowed HS-QAF-AgNPs hydrogel with enhanced bacteriostatic effects. *In vitro* results showed that the HS-QAF-AgNPs hydrogel significantly promoted cell migration and angiogenesis, making it a promising candidate for wound healing applications. Moreover, the HS-QAF-AgNPs hydrogel demonstrated a potent capacity to mitigate the inflammatory response. Mechanistically, it exerted its anti-inflammatory effects by obstructing the activation of the NF-κB signaling cascade, augmenting the expression of the anti-inflammatory cytokine IL-10, and reducing the secretion of the pro-inflammatory cytokines TNF-α and IL-6. The outstanding *in vitro* anti-inflammatory properties, cellular behavior, and antibacterial efficacy motivate us to further explore and achieve effective wound healing outcomes *in vivo* using infected full-thickness scald wound model. HS-QAF-AgNPs hydrogel could induce the orderly deposition of collagen, delay apoptosis, promote cell proliferation and significantly accelerate the healing of severe scalds. In summary, the developed HS-QAF-AgNPs hydrogel dressings represents a versatile treatment in infectious wound management and could be investigated as ideal candidates for advanced wound dressings.

## Data Availability

The original contributions presented in the study are included in the article/Supplementary material, further inquiries can be directed to the corresponding authors.
