# Supplementary material for: Exosome microsphere/nano silver loaded injectable antibacterial hydrogel augments anti-infection and healing for scald wound
Source: Front Microbiol. 2025 Jun 25;16:1550276. doi: 10.3389/fmicb.2025.1550276 (PMC12238018; doi:10.3389/fmicb.2025.1550276)
Supplement: Supplementary file 1 [file Data_Sheet_1.docx]

**Exosome** **microsphere/nano silver loaded injectable antibacterial hydrogel augments anti-infection and healing for scald wound**

**1. Injectable and self-healing property**

Rheological tests were carried out on HS-QAF-AgNPs hydrogels using a TA rheometer (Newcastle DHR-1, USA). The hydrogels placed onto the rheometer were formed into cylindrical discs with a thickness of 0.5 mm and a diameter of 40 mm. Time sweep measurements were carried out at a constant frequency of 1 Hz and a strain of 1% at room temperature to evaluate the storage modulus (G′) and loss modulus (G″) of the hydrogels. The time sweep range was from 0 s to 300 s. The TA rheometer was used to measure the critical strain by varying the strain amplitude from 1% to 1000%. Alternate step strain sweep measurements were conducted at a constant angular frequency of 1 Hz. The amplitude oscillation strains were alternated from small strain (γ = 1%, 60 s for each interval) to large strain (γ = 1000%, 60 s for each interval). Cylindrical hydrogels discs with a thickness of 8 mm and a diameter of 10 mm were created. The hydrogels were split into two sections, and then brought together at 37°C without any external stimulation. Subsequently, the ability of the hydrogels to support their own weight was assessed by lifting them. The continuous ﬂow experiments (shear rates from 0.01 to 100 s^-1^) were conducted to measure the shear viscosity of the hydrogels.

The injectability of the hydrogel was also evaluated through macroscopic experiments. Specifically, a solution containing exo-CAMs, AgNPs, and QAF hydrogel was uniformly mixed in the syringe. The resulting mixture was drawn into a 5 mL syringe and then extruded onto a glass plate. Bromine thymol blue indicator was utilized as a stain. To assess the flow properties of the injection, we inscribed "BZMU" on the glass plate.

**2. Swelling and water uptake properties**

The hydrogels were freeze-dried and shaped at room temperature before being weighed (*m*_0_). Their swelling properties were assessed by immersing them in PBS (pH 7.4) until they reached a state of equilibrium. After absorbing surface moisture with filter papers, the fully swelled hydrogels were weighed (*m_t_*). Each experiment was repeated three times. The water uptake rate was then calculated using equation .

**3. Porosity of hydrogel samples**

For the porosity test, the freeze-dried QAF and HS-QAF-AgNPs hydrogel were weighed (*V*_0_) and immersed in anhydrous ethanol overnight. The following day, the samples were removed from the ethanol and the excess ethanol was carefully wiped away with filter paper before being weighed (*V_t_*). Each experiment was repeated three times. The porosity of hydrogels was calculated using equation .

**4.** **CD63 Elisa kit**

1 mL HS-QAF-AgNPs composites was placed at 37°C incubator for 30 min to form stable gels. The gels were incubated with 1 mL PBS in a constant-temperature shaker at 37 °C. After specific time intervals, 0.5 mL of the supernatant was collected for analysis, and an equal volume of fresh PBS was added for further incubation. The quantity of released huMSCs-exos in the supernatants was measured using a Human CD63 Elisa kit according to the manufacturer's instructions.

**5. Hemolysis, blood clotting and liver hemostasis experiment**

The mouse blood was centrifuged at 2500 rpm for 10 min to obtain the erythrocytes. Hydrogel (100 µL) mixed with erythrocytes stock (500 µL) and PBS (500 µL) was incubated at 37 °C for 1 h. After centrifugation at 1000 rpm for 15 min, the supernatant (100 µL) from each group was added to a 96-well microplate. The OD values were measured at a wavelength of 545 nm using a microplate reader. According to the standard ISO 10993-4:2017, a hemolysis rate of less than 5% is considered to meet the requirement for good hemocompatibility. The positive control used was 0.1% Triton x-100, while the negative control used was PBS buffer. Each experiment was repeated three times. The hemolysis rate was calculated using equation .

$$\text{Hemolysis rate (\%)=}\frac{OD_{x} -OD_{p} }{OD_{t} -OD_{p} }\times100\%$$

where *OD_x_*, *OD_p_*, and *OD_t_* were the OD values for the experimental group, PBS buffer negative control, and 0.1% Triton x-100 positive control, respectively.

100 μL of blood from mouse eyeballs were mixed with the prepared hydrogels, and then 10 μL of a 0.2 M solution of CaCl_2_ was added. The mixture was then kept at 37 °C for 10 min. After that, the hydrogels were immersed in deionized water and their absorbance at 540 nm was measured. The absorbance of whole blood in deionized water served as a reference. The experiment was repeated three times under the same conditions. Each experiment was repeated three times. The blood clotting index (BCI) of the samples was determined using equation .

After being anesthetized, a mouse was secured onto a surgical corkboard. The liver was removed through an abdominal incision and placed on filter paper. A needle was used to induce bleeding from the liver, followed by the application of a hydrogel solution to the bleeding site. Two minutes later, the weight of the blood-absorbed filter paper was measured and compared with a control group that did not undergo any treatment after liver puncture.

**6 *In Vitro* Cell migration** **and Tube Formation**

Cell migration was studied using a scratch assay. HaCaT cells were seeded in 6-well plates at a 1.2 × 10^5^ cells per well. Five parallel scratches were created in each well using a sterlized 200 μL plastic pipette tip, and the width of each scratch was measured as the baseline value. The cells were then incubated in different groups in a humidified atmosphere containing 5% CO_2_ for 24, 48, and 72 h. The width of the scratch was visualized using a light microscope (Leica DM 2500, Germany) and quantified by employing an exclusion zone assay method utilizing ImageJ software.

For the transwell assay, HUVECs (4 × 10^4^ cells/chamber) were added to the upper chamber and the lower champer was filled with QAF or HS-QAF-AgNPs hydrogel extract. The remaining cells in the upper chamber were gently wiped off with a sterile cotton swab after incubation at 37°C for 24 h. the migrated cells were fixed with 4% paraformaldehyde for 20 min and then stained with crystal violet for 15 min. Finally, images of the cells were taken using an inverted phase contrast microscope (Olympus CKX53, Japan), and then the crystal violet was dissolved in a 10% acetic acid solution. The OD value of the solution was measured at 595 nm to analyze the number of migrated cells.

For the tube formation assay, the HUVECs (1 × 10^5^ cells per well) were seeded onto a Matrigel-coated plate and treated with 100 μL extracts of QAF and HS-QAF-AgNPs hydrogel. After incubation at 37 °C under 5% CO_2_ for 6 h, the cells were imaged using a microscope (Olympus CKX53, Japan). The total tube length and branch points were calculated using ImageJ software (NIH, Bethesda, MD, USA).

**7 *In Vitro* Anti-Inflammation Activity**

The *in vitro* model of inflammatory cells was initially established, using RAW 264.7 mouse mononuclear macrophages as the model cells. Cell supernatant was aspirated, centrifuged at 1000 rpm for 5 min, and transferred for secondary centrifugation. After washing the RAW 264.7 cells once with PBS solution, RIPA lysate was added to extract protein which was then boiled with 5X buffer for 10 minutes and stored at -20℃ for later use.

Equal-quality cell extracts were subjected to sodium dodecyl sulfate–polyacrylamide gel electrophoresis (SDS-PAGE) on 10% polyacrylamide gels, followed by transfer to PVDF membranes and blocking with 5% BSA in TBS–Tween 20 (TBST). Primary antibodies were then applied to the membranes overnight at 4°C. After washing, a secondary antibody was used for probing the membranes for 1 hour at room temperature, followed by treatment with SuperSignal West Pico Chemiluminescent Substrate. Chemiluminescent visualization was carried out according to the ECL color kit instructions in the dark.

The concentrations of the inflammatory cytokines IL-6 and TNF-α, as well as the anti-inflammatory cytokine IL-10, were measured in the cell supernatant obtained in the step following the instructions provided by the ELISA kit.

**8. *In vitro*** **hemocompatibility and cytocompatibility evaluation**

The CCK-8 assay was used to evaluate cell proliferation. Hydrogels were immersed in fresh DMEM medium at 37°C for 24 h to prepare an extract. L929 fibroblasts from three generations were seeded onto 96-well plates (8 × 10^3^ cells per well) in DMEM medium. The plates were then incubated in a humidified atmosphere with 5% CO_2_ at 37°C for 24 h. Following this, the original DMEM medium was replaced with the extracts, while blank wells were used as controls and their mediums refreshed. CCK-8 reagent was added to each well and incubated further at 37°C for 1 h, after which the OD value was measured at 450 nm. Each experimental group was assayed in six technical replicates. Cell viability was then calculated using equation .

$$\text{Cell viability }(\%)=\frac{OD_{sample} }{OD_{\text{control}} }\times\text{100\%}$$

**9. The release of hucMSCs-exos**

1 mL of HS-QAF-AgNPs complex (containing 100 mg exo-CAMs) and 100 mg exo-CAMs were incubated with 2 mL of PBS in a constant-temperature shaker at 37 ℃. At predetermined time intervals, supernatant (1 mL) was collected for analysis and replaced with an equivalent volume of fresh PBS (1 mL) to maintain incubation continuity. The released human mesenchymal stem cell-derived exosomes in the supernatant were quantified using Coomassie Brilliant Blue G250 (EHC108.96.5 kit, Neobioscience, China) following the manufacturer's protocol.

**10. Supplementary results**


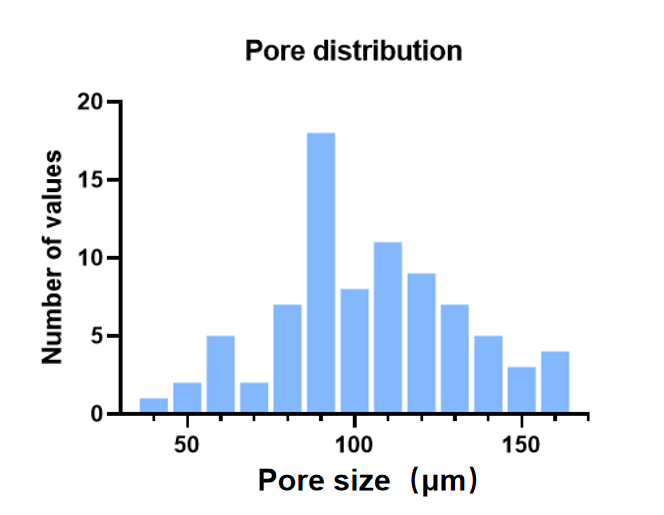


**Figure S1** Pore size of HS-QAF-AgNPs Hydrogel.


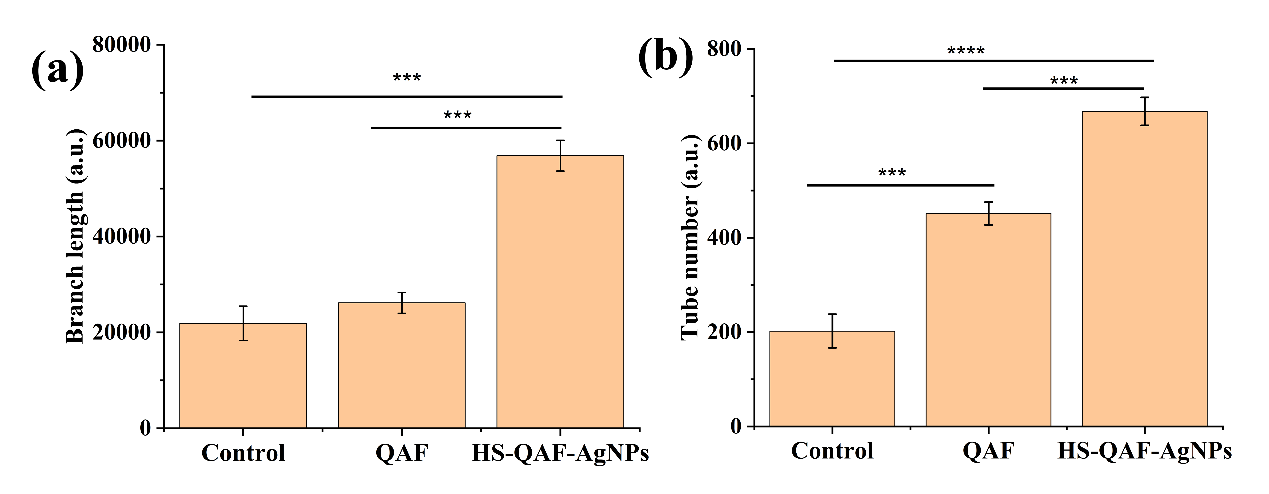


**Figure S2** Branch length (a) and tube number (b) in the tube formation assay. ****p* < 0.001, *****p* < 0.0001, n = 3.

**References**

[1] H. Yan, C. Wang, Q. Zhang, P. Yu, Y. Xiao, C. Wang, P. Zhang, G. Hou, Conductive Polyaniline Particles Regulating In Vitro Hydrolytic Degradation and Erosion of Hydroxyapatite/Poly(lactide-co-glycolide) Porous Scaffolds for Bone Tissue Engineering, ACS Biomater. Sci. Eng., 9 (2023) 1541-1557.

[2] X. Xie, J. Cai, D. Li, Y. Chen, C. Wang, G. Hou, T. Steinberg, B. Rolauffs, M. El-Newehy, H. El-Hamshary, J. Jiang, X. Mo, J. Zhao, J. Wu, Multiphasic bone-ligament-bone integrated scaffold enhances ligamentization and graft-bone integration after anterior cruciate ligament reconstruction, Bioact. Mater., 31 (2024) 178-191.

[3] C. Wang, Q. Zhang, G. Hou, C. Wang, H. Yan, Sustained release of EGF/bFGF growth factors achieved by mussel-inspired core–shell nanofibers with hemostatic and anti-inflammatory effects for promoting wound healing, European Polymer Journal, 190 (2023) 112003.

[4] Y.-L. Zhang, C. Wang, X.-Q. Yuan, H.-H. Yan, C.-B. Li, C.-H. Wang, X.-R. Xie, G.-G. Hou, Multifunctional xyloglucan-containing electrospun nanofibrous dressings for accelerating infected wound healing, Int. J. Biol. Macromol., 247 (2023) 125504.

[5] B.M. Bakadia, A.A. Qaed Ahmed, L. Lamboni, Z. Shi, B. Mutu Mukole, R. Zheng, M. Pierre Mbang, B. Zhang, M. Gauthier, G. Yang, Engineering homologous platelet-rich plasma, platelet-rich plasma-derived exosomes, and mesenchymal stem cell-derived exosomes-based dual-crosslinked hydrogels as bioactive diabetic wound dressings, Bioact. Mater., 28 (2023) 74-94.
